# Global adolescent self-harm (10–19 years): 1990–2021 trends, health inequalities, frontier analysis, and 2035 projections using global burden of disease data

**DOI:** 10.3389/fpubh.2026.1689706

**Published:** 2026-02-03

**Authors:** Hui Zhang, Tulips Yiwen Wang, Jiang Nan, Hongjuan Jiang, Sheau Tsuey Chong, Zheng Wang, Jing Guo, Chunyi Chen

**Affiliations:** 1 UWE College (School of International Education), Hainan Medical University, Haikou, Hainan, China; 2 Faculty of Health and Wellness, City University of Macau, Macau, China; 3 Department of Psychology, Hainan Medical University, Haikou, Hainan, China; 4 Centre for Research in Psychology and Human Well-Being, Faculty of Social Sciences and Humanities, Universiti Kebangsaan Malaysia, Bangi, Malaysia; 5 Counselling Psychology Program, Postgraduate Secretariat, Faculty of Social Sciences and Humanities, Universiti Kebangsaan Malaysia, Bangi, Malaysia; 6 School of Public Health, Hainan Medical University, Haikou, Hainan, China

**Keywords:** adolescents, average annual percent change (AAPC), global burden of disease, self-harm, socio-demographic index (SDI), years lived with disability (YLDs)

## Abstract

**Objectives:**

Adolescent self-harm is a major global health concern, yet evidence focused specifically on those aged 10–19 years remains limited. This study comprehensively assessed its global burden.

**Methods:**

Using data from the Global Burden of Disease Study 2021, we analyzed trends in incidence, years lived with disability (YLDs), disability-adjusted life years (DALYs), and risk factors from 1990 to 2021, and projected trends to 2035. Frontier analysis, health inequality assessment, and autoregressive integrated moving average (ARIMA) models were applied, and average annual percent change (AAPC) was estimated for 204 countries and territories.

**Results:**

In 2021, the global incidence of adolescent self-harm was 66.75 per 100,000 (95% CI: 43.34 to 97.12). Females showed 1.85-fold and 1.82-fold higher incidence and YLDs rates than males, while males had 1.16-fold higher DALYs rates. Greenland ranked highest across all three rates. From 1990 to 2021, incidence, YLDs, and DALYs rates declined in absolute terms, yet 91 countries exhibited increasing AAPC values (relative rise). The incidence and YLDs rates were significantly associated with the socio-demographic index (SDI), while DALYs were not. The Slope Index of Inequality decreased to −17.74, reflecting a growing concentration of burden in low-SDI settings, whereas some high-SDI settings showed persistent health-efficiency gaps. The population attributable fractions of high alcohol use and high temperature increased to 2.82 and 2.80%, respectively. Forecasts suggest declining incidence and DALYs rates but a rising YLDs rates by 2035.

**Conclusion:**

Nearly half of all countries show rising trends in adolescent self-harm incidence, with burdens shifting toward non-fatal outcomes and low-SDI settings, underscoring the need for gender-sensitive, equity-focused, and prevention-oriented global strategies to guide future self-harm intervention policies.

## Introduction

Self-harm is defined as the deliberate act of inflicting direct physical injury to body tissue (1, 2). The World Health Organization defines individuals aged 10–19 as “adolescents” (3). Adolescence (10–19 years) is a critical period of rapid physical, cognitive, and emotional development (4). Globally, about 17% of adolescents have engaged in self-harm (5), making it a major public health concern (6). Self-harm behaviors range from non-suicidal self-harm to suicide, reflecting the interaction of multiple psychological, social, and environmental factors (7–9). The methods vary, including overdose, cutting, burning, and head banging (10). Among adolescents hospitalized for self-harm, one in 200 dies within 3 years, and the recurrence rate of non-fatal self-harm reaches 27.3% (11, 12). These data underscore the urgency of early identification and prevention.

Global meta-analyses and Global Burden of Disease (GBD) studies indicate that the burden of self-harm is highest among those aged 15–49 (13, 14), with a higher incidence of non-fatal self-harm in females (13, 15). The burden is greater in low- and middle-income regions (15), whereas self-harm mortality among male adolescents is higher in high-SDI settings (16), highlighting heterogeneity across populations and contexts. Despite several studies using GBD data^1^ to analyze the epidemiological characteristics of self-harm, several key limitations persist (17). First, most large-scale analyses have targeted broader age ranges (e.g., 10–24 years) or combined self-harm with related topics such as substance use and interpersonal violence (9, 13, 16, 18). However, adolescents aged 10 to 19 show significant differences in neurocognitive development compared to young adults aged 20 to 24 (19), as well as differences in the onset and peak of self-harming behaviors, which typically begin around age 13 and peak between 15 and 17 (20, 21). Combining these groups may therefore obscure the distinct epidemiological patterns of adolescent self-harm. Second, current GBD-based research has primarily emphasized fatal burden measured by disability-adjusted life years (DALYs), with insufficient focus on non-fatal health loss, represented by years lived with disability (YLDs). This gap limits the understanding of the long-term disabling consequences of self-harm. Given that adolescence is a critical period for prevention and intervention (17), studies focusing exclusively on individuals aged 10–19 years and jointly addressing YLDs and DALYs remain limited. Finally, few studies have integrated frontier analysis, health inequality metrics, such as the Slope Index of Inequality (SII) and Concentration Index (CI), and time-series forecasting models, such as ARIMA. The lack of such multidimensional approaches constrains the comprehensive understanding of how self-harm burden interacts with socioeconomic development, health system efficiency, and its potential future trajectories.

To fill these gaps, this study used GBD 2021 data to analyze the global burden of self-harm among adolescents aged 10–19 years across 204 countries and territories from 1990 to 2021. We assessed both non-fatal (YLDs) and fatal (DALYs) burdens and explored their associations with socioeconomic development and health efficiency. Specifically, this study aimed to (1) compare sex- and region-specific differences in self-harm incidence, YLDs, and DALYs; (2) calculate the average annual percent change (AAPC) to identify countries with increasing trends; (3) examine the relationship between self-harm burden and the socio-demographic index (SDI); (4) evaluate health efficiency gaps using frontier analysis; (5) assess the attributable burden of behavioral and environmental risk factors; (6) project future trends to 2035.

## Methods

### Conceptual framework

Guided by the social ecological perspective and the stress–diathesis model (22–24), we posited that upstream structural context shapes adolescent self-harm burden through proximal exposures and access to prevention and care, with sex as a potential effect modifier. This framework guided variable selection, stratification, and interpretation.

### Data sources

Self-harm in the GBD framework is defined as intentional self-inflicted injury to body tissue, encompassing both nonfatal and fatal outcomes (25). Codes were classified according to ICD-9 (E950–E959) and ICD-10 (X60–X64.9, X66–X84.9, Y87.0) (2).

Data for this study were obtained from GBD 2021, which provides standardized estimates of incidence, DALYs, and YLDs for 204 countries and territories. GBD data are synthesized from multiple sources, including vital registration, hospital records, and household surveys, and are modeled using the Bayesian meta-regression tool DisMod-MR 2.1. All estimates were reported with 95% uncertainty interval (UI) derived from 1,000 posterior draws (26).

Incidence rate reflects the number of new cases of self-harm among adolescents within a specific time period, measuring the risk of self-harm and the effectiveness of prevention. YLDs quantify the burden of non-fatal health outcomes by weighting the duration of disability according to the severity of the condition. YLDs were chosen to capture the non-fatal component and long-term functional impairment of adolescent self-harm. DALYs combine premature death-related life loss (Years of life lost, YLL) and non-fatal loss (YLD) to measure the overall burden of self-harm on adolescents’ overall health (26). AAPC assesses the overall trend of self-harm burden during the study period, capturing long-term dynamic patterns and supporting cross-national comparisons (27). SII and CI reflect the absolute and relative inequalities in disease burden across different socioeconomic levels, providing a basis for identifying vulnerable groups and assessing progress toward health equity (28, 29). SDI, obtained from the Institute for Health Metrics and Evaluation (IHME), is a composite measure combining per capita income, average years of schooling among individuals aged ≥15 years, and total fertility rate among those aged <25 years. It indicates a country’s socioeconomic development level and helps assess self-harm burden across development stages. According to the GBD 2021 classification, countries and territories were grouped into five SDI quintiles (high, high-middle, middle, low-middle, and low). Countries in the highest and lowest quintiles were classified as the high-SDI and low-SDI groups, respectively. SDI was applied both as a categorical variable for group comparisons and as a continuous variable to explore correlations with self-harm burden (26). Self-harm-related risk factors in GBD 2021 include drug use, alcohol consumption, and exposure to high and low temperatures (26). The population attributable fraction (PAF) was used to quantify the proportion of self-harm burden that could be averted by eliminating specific risk exposures (30). Estimates with wide UIs were retained but interpreted cautiously, as the Bayesian framework of GBD inherently accounts for data sparsity and low-confidence inputs through posterior sampling (31). All rates were age-specific for adolescents aged 10–19 years (per 100,000 population).

Data were downloaded from the Global Health Data Exchange (GHDx) in CSV format (accessed December 1, 2024). Detailed sources can be accessed via the GBD 2021 Data Input Sources Tool. GBD 2021 was approved by the Institutional Review Board of the University of Washington. This study used aggregated and publicly available data, thus, no additional ethical approval was required.

### Geographical locations reported

This study analyzed all 204 countries and territories covered by GBD 2021, which include diverse populations at different SDI levels (32). To ensure comparability across 204 countries and territories, this study did not employ external systems to group countries by additional social, economic, or cultural characteristics, as these classification methods do not fully correspond to the geographic scope of GBD. The results were aggregated globally to reflect overall burden patterns without further sub-regional stratification.

## Statistical analysis

### Trend analysis

Temporal trends in adolescent self-harm burden from 1990 to 2021 were assessed using the AAPC, which summarizes overall rate changes across the study period and enables comparability across countries and metrics. Analyses were conducted in R (version 4.4.2) using the segmented package. A generalized linear model was fit with calendar year as the independent variable and the natural logarithm of age-specific rates as the dependent variable. Piecewise regression was then used to estimate trend breakpoints and segment slopes. AAPC was calculated as a weighted average of segment-specific annual percent changes (APCs), weighted by the duration of each segment. The 95% confidence interval (95% CI) for the AAPC were derived using the Delta method to account for both sampling error and model uncertainty (33, 34).

### Health inequality analysis

To evaluate socioeconomic inequalities in adolescent self-harm burden, both the SII and CI were calculated (35). In this manuscript, “CI” refers to the Concentration Index, whereas “95% CI” denotes the 95% confidence interval. SII measures the absolute difference in burden between the highest and lowest SDI groups via weighted linear regression (SII > 0 indicates a burden concentrated in high-SDI countries; SII < 0 indicates concentration in low-SDI countries) (36). CI quantifies relative inequality based on the Lorenz curve principle, ranging from −1 to +1 (CI = 0 denotes perfect equality; CI < 0 pro-poor; CI > 0 pro-rich) (29). Together, these indices capture both absolute and relative dimensions of socioeconomic inequality in adolescent self-harm burden.

### Frontier analysis

To assess the performance of national health systems in mitigating adolescent self-harm burden relative to development level, frontier analysis was conducted using data envelopment analysis. This approach identifies the best-achievable frontier of minimum DALY rates for a given SDI level (37). The vertical distance between observed and frontier values was defined as the efficiency gap. Uncertainty was addressed through 1,000 bootstrap replications to derive 95% CIs and ensure robust estimates.

### Forecasting analysis

Future trends (2022–2035) were projected using the autoregressive integrated moving average (ARIMA) model. Model parameters (p, d, q) were selected using the *auto.arima* function in R (v4.4.2) is based on the KPSS stationarity test and the minimum AIC/BIC criteria. Model diagnostics included (1) KPSS test for stationarity, (2) Ljung–Box Q test for residual independence, (3) ACF/PACF plots for autocorrelation inspection (38, 39). Residual plots were further examined to confirm the absence of heteroscedasticity and autocorrelation, ensuring goodness of fit.

### Software

All analyses and visualizations were performed using R (version 4.4.2) and JD_GBDR (version 2.7.4).

## Results

### Global self-harm burden among adolescents aged 10–19 years in 2021

In 2021, the number of adolescents who self-harm accounted for 15.70% of all self-harm cases globally. The global incidence rate was 66.75 per 100,000 (95% UI: 43.34–97.12), and the YLDs and DALYs rates were 2.22 (95% UI: 1.44–3.17) and 284.35 (95% UI: 260.50–317.98) per 100,000, respectively. Females had 1.85-fold and 1.82-fold higher incidence and YLDs rates than males, while males had 1.16-fold higher DALYs rates, reflecting a higher frequency of self-harm in females but a higher fatal burden in males (Table 1).

**Table 1:** Global self-harm incidence, YLDs, and DALYs: numbers and age-specific rates among all ages and adolescents aged 10–19 years, by sex, in 2021.

Age group	Measure	Sex	Number (1000’s) (95% UI)	Rate per 100,000 (95% UI)
10–19 years	Incidence	Both	861.42 (559.31–1,253.50)	66.75 (43.34–97.12)
		Female	547.81 (354.67–797.22)	87.43 (56.60–127.23)
		Male	313.62 (202.94–453.53)	47.23 (30.56–68.30)
	YLDs	Both	28.63 (18.60–40.88)	2.22 (1.44–3.17)
		Female	18.09 (11.70–26.11)	2.89 (1.87–4.17)
		Male	10.55 (6.99–14.91)	1.59 (1.05–2.25)
	DALYs	Both	3,669.92 (3,362.01–4,103.88)	284.35 (260.50–317.98)
		Female	1,645.40 (1,448.98–1,877.59)	262.60 (231.25–299.66)
		Male	2,024.52 (1,841.91–2,262.94)	304.88 (277.38–340.78)
All ages	Incidence	Both	5,487.77 (4,598.83–6,504.69)	69.54 (58.28–82.43)
		Female	3,274.64 (2,730.87–3,909.14)	83.28 (69.45–99.42)
		Male	2,213.12 (1,858.50–2,592.30)	55.90 (46.94–65.47)
	YLDs	Both	898.35 (639.52–1,187.43)	11.38 (8.10–15.05)
		Female	542.27 (390.59–722.80)	13.79 (9.93–18.38)
		Male	356.07 (256.72–469.49)	8.99 (6.48–11.86)
	DALYs	Both	33,525.09 (31,307.62–35,841.26)	424.83 (396.73–454.18)
		Female	10,512.45 (9,339.38–11,849.30)	267.36 (237.52–301.36)
		Male	23,012.64 (21,582.79–24,602.18)	581.22 (545.10–621.36)

Data are presented as rates with 95% uncertainty interval (UI). Years Lived with Disability; DALYs, Disability-Adjusted Life Years. Numbers are presented in thousands (1000’s). Rates are per 100,000 population. Source: Global Burden of Disease Study 2021 (Institute for Health Metrics and Evaluation, IHME).

**Figure 1:**
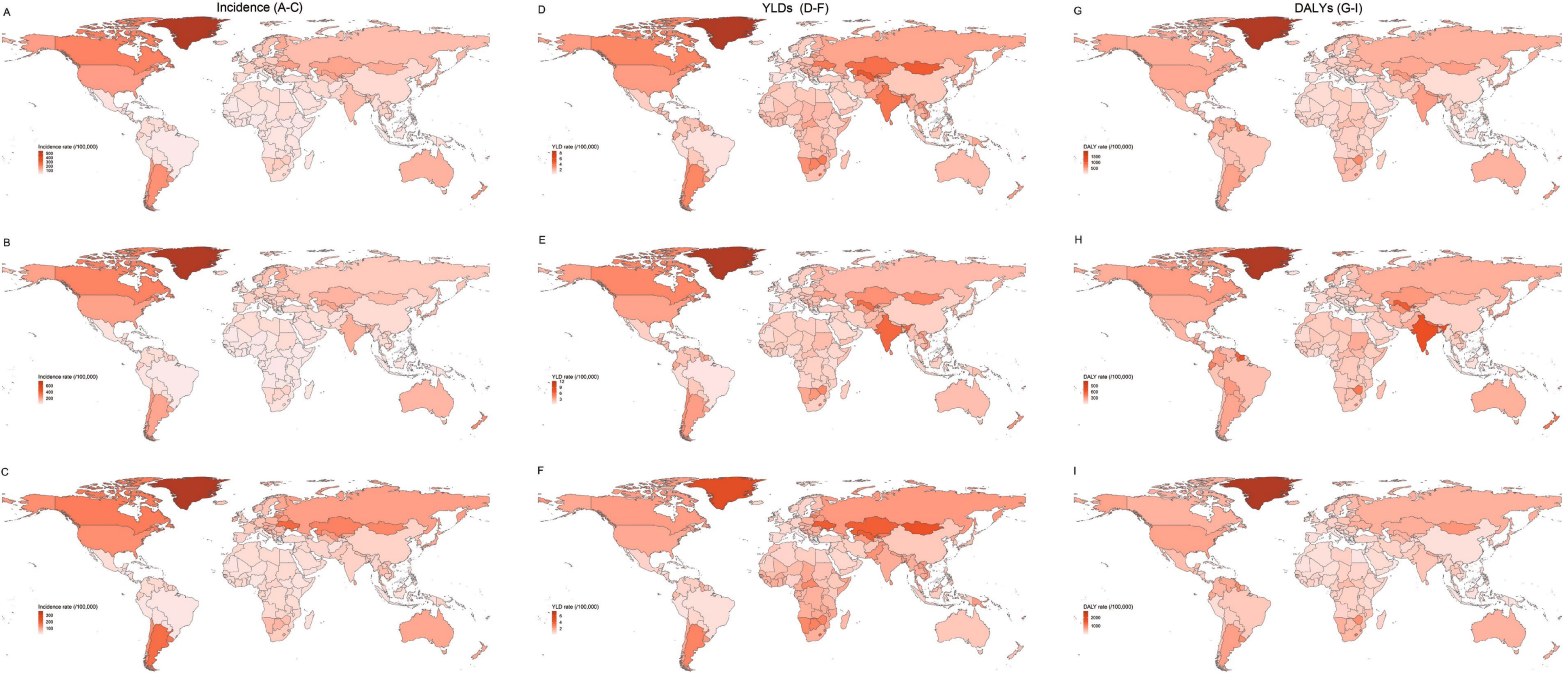
Global distribution of self-harm incidence, YLDs, and DALYs rates (per 100,000 population) among adolescents aged 10–19 years across 204 countries and territories in 2021. Left column **(A–C)**: Incidence rates for both sexes **(A)**, females **(B)**, and males **(C)**. Middle column **(D–F)**: YLDs rates for both sexes **(D)**, females **(E)**, and males **(F)**. Right column **(G–I)**: DALYs rates for both sexes **(G)**, females **(H)**, and males **(I)**. YLDs, years lived with disability; DALYs, disability-adjusted life years.

Geographically, the adolescent self-harm burden was highly uneven. Greenland ranked first globally in incidence, YLDs, and DALYs rates, emerging as the hotspot (Figures 1A,D,G; Supplementary Table 1). By sex, Greenland females had the highest incidence and YLDs rates, while Palau females had the highest DALYs (Figures 1B,E,H; Supplementary Table 1). Among males, Kiribati showed the highest YLDs, and Greenland the highest incidence and DALYs (Figures 1C,F,I; Supplementary Table 1). These were mainly located in Pacific islands and extreme environments, suggesting that environmental vulnerability may be an important risk factor.

### Temporal and spatial trends in self-harm burden among adolescents aged 10–19 years, 1990–2021

**Figure 2:**
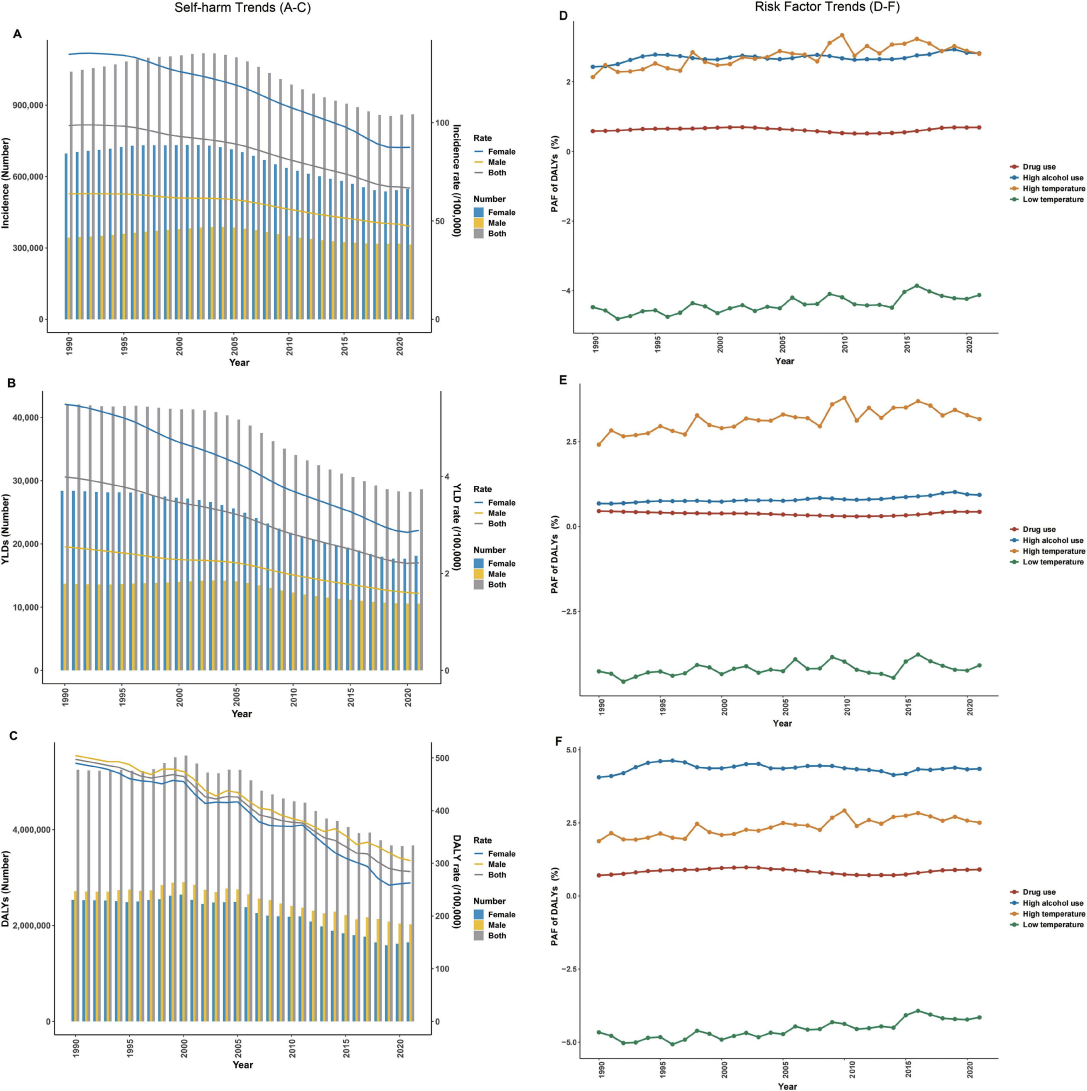
Temporal trends in the self-harm burden and attributable risk factors among adolescents aged 10–19 years from 1990 to 2021. Left column **(A–C)**: Trends in age-specific incidence **(A)**, YLDs **(B)**, and DALYs rates **(C)** for self-harm. Right column **(D–F)**: Temporal changes in population attributable fractions (PAF) for attributable risk factors—including drug use, high alcohol use, high temperature, and low temperature for both sexes **(D)**, females **(E)**, and males **(F)**. PAF, population attributable fraction; YLDs, years lived with disability; DALYs, disability-adjusted life years.

From 1990 to 2021, the global age-specific rates of self-harm among adolescents aged 10–19 years exhibited an overall downward trend. Throughout the period, females consistently exhibited higher incidence and YLDs rates than males (Figures 2A,B; Supplementary Table 2), whereas males had persistently higher DALYs rates (Figure 2C; Supplementary Table 2).

**Figure 3:**
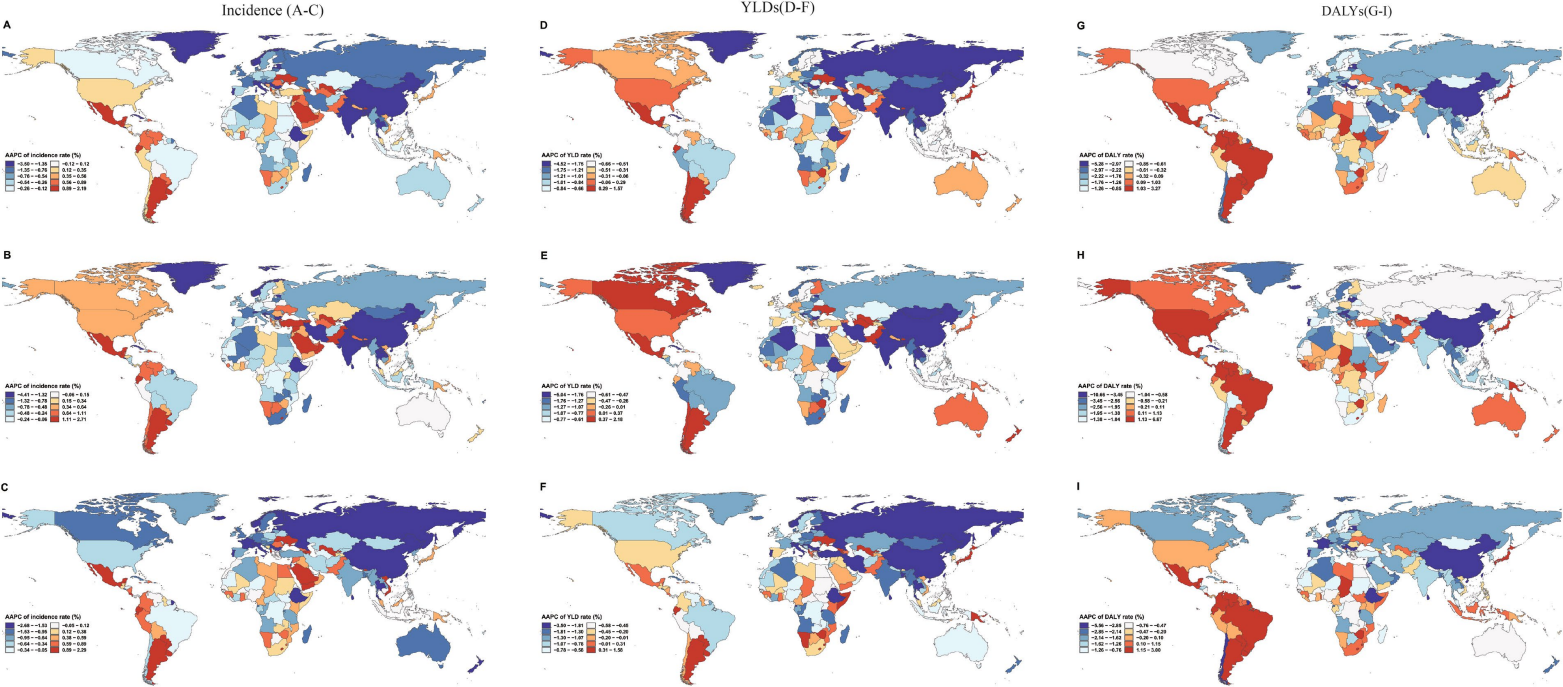
Global average annual percentage change (AAPC) in self-harm incidence, YLDs, and DALYs rates (per 100,000 population) among adolescents aged 10–19 years across 204 countries and territories, 1990–2021. Left column **(A–C)**: AAPC in incidence rates for both sexes **(A)**, females **(B)**, and males **(C)**. Middle column **(D–F)**: AAPC in YLDs rates for both sexes **(D)**, females **(E)**, and males **(F)**. Right column **(G–I)**: AAPC in DALYs rates for both sexes **(G)**, females **(H)**, and males **(I)**. AAPC, average annual percentage change; YLDs, years lived with disability; DALYs, disability-adjusted life years.

However, considerable heterogeneity was observed across countries and territories. Regarding incidence rates, 91 out of 204 countries and territories (44.61%) recorded increasing trends (AAPC > 0), highlighting that global progress was uneven and that nearly half of the world continued to experience a growing burden of adolescent self-harm. The largest increase was observed in Uzbekistan (AAPC = 2.16, 95% CI: 2.06 to 2.26), whereas the steepest decline occurred in Cuba (AAPC = −3.46, 95% CI: −3.60 to −3.33) (Figure 3A; Supplementary Table 3). Sex-stratified analysis revealed that incidence increased in 98 countries among females and 92 countries among males, with the steepest increases observed in Uzbekistan (females) and Ecuador (males; Figures 3B,C; Supplementary Table 3).

For YLDs rates, 33 countries and territories displayed upward trends, with Uzbekistan showing the largest increase (AAPC = 1.56, 95% CI: 1.39 to 1.73) and China the largest decrease (AAPC = −4.47, 95% CI: −4.80 to −4.14; Figure 3D; Supplementary Table 3). Similar patterns were observed by sex, with 41 countries among females and 38 among males exhibiting increasing YLDs trends; the steepest increases were found in Uzbekistan (females) and Argentina (males; Figures 3E,F; Supplementary Table 3).

For DALYs rates, 44 countries and territories experienced increasing trends, with Tokelau showing the largest increase (AAPC = 4.61, 95% CI: 2.21 to 7.01) and China showing the greatest decline (AAPC = −5.23, 95% CI: −5.50 to −4.95; Figure 3G; Supplementary Table 3). Among females and males, 46 and 48 countries, respectively, showed increasing DALYs trends, with Niue and Tokelau (females) and Tokelau (males) exhibiting the most rapid increases (Figures 3H,I; Supplementary Table 3). Overall, although the global burden of adolescent self-harm has declined since 1990, it has become increasingly concentrated in specific high-burden areas.

### Relationship between self-harm burden and SDI across 204 countries and territories

**Figure 4:**
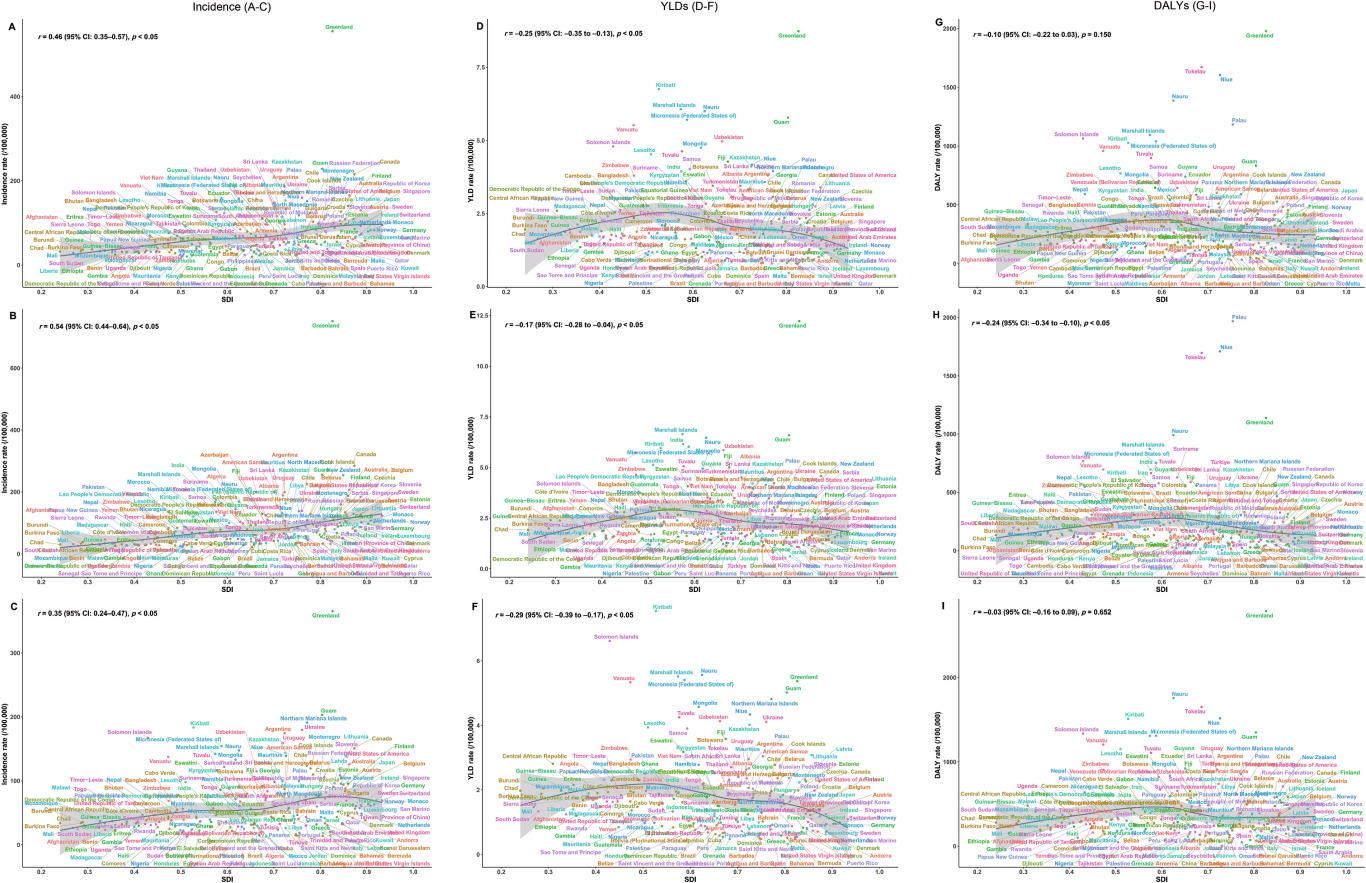
Associations between self-harm burden and the socio-demographic index (SDI) among adolescents aged 10–19 years across 204 countries and territories in 2021, stratified by sex. Left column **(A–C)**: Incidence rates for both sexes **(A)**, females **(B)**, and males **(C)**. Middle column **(D–F)**: YLDs rates for both sexes **(D)**, females **(E)**, and males **(F)**. Right column **(G–I)**: DALYs rates for both sexes **(G)**, females **(H)**, and males **(I)**. SDI, Socio-demographic Index; YLDs, years lived with disability; DALYs, disability-adjusted life years.

The associations between SDI and adolescent self-harm burden varied by metric and sex. In 2021, the incidence rates of self-harm showed a significant positive correlation with SDI (both sexes: *r* = 0.46; females: *r* = 0.54; males: *r* = 0.35; all *p* < 0.05; Figures 4A–C), suggesting that countries and territories with higher levels of social development tend to report higher self-harm incidence. In contrast, YLDs rates were negatively correlated with SDI (both sexes: *r* = −0.25; females: *r* = −0.17; males: *r* = −0.29; all *p* < 0.05; Figures 4D–F), indicating that although self-harm is more frequently reported in high-SDI settings, its nonfatal health burden is relatively lower. The relationship between DALYs rates and SDI differed by sex. A significant negative correlation was observed only among females (*r* = −0.24, *p* < 0.05) (Figure 4H), while no significant associations were found for both sexes combined (*r* = −0.10) or for males (*r* = −0.03; both *p* > 0.05; Figures 4G,I). Suggesting that females in higher-SDI settings have achieved greater reductions in fatal self-harm outcomes than males.

### Health inequality in adolescent self-harm across 204 countries and territories, 1990–2021

**Figure 5:**
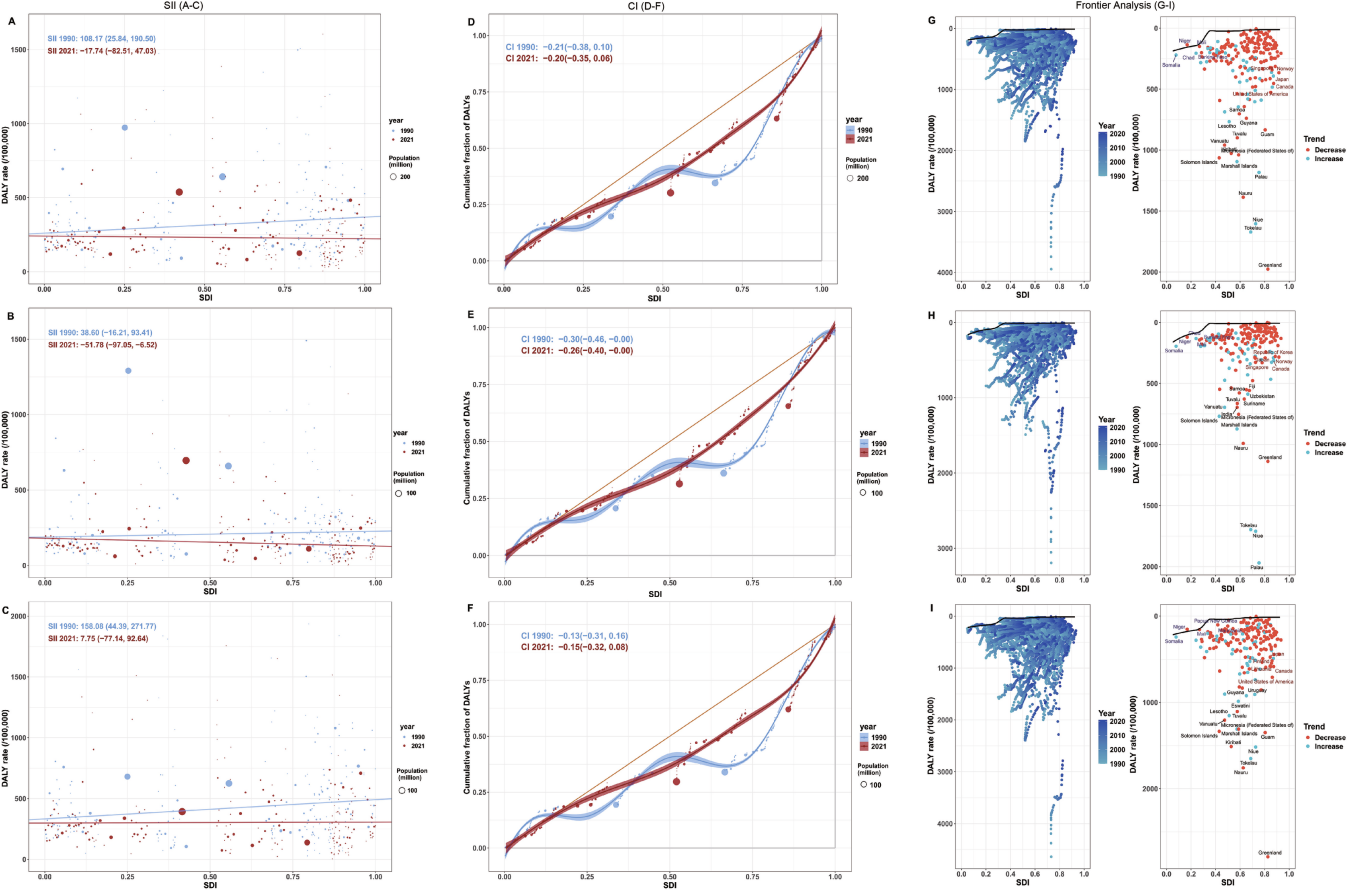
Socioeconomic inequality and frontier analysis of self-harm DALYs rates among adolescents aged 10–19 years across 204 countries and territories, 1990–2021. Left column **(A–C)**: Slope index of inequality (SII) for self-harm DALYs in 1990 and 2021 for both sexes **(A)**, females **(B)**, and males **(C)**. Middle column **(D–F)**: Concentration index (CI) for self-harm DALYs in 1990 and 2021 for both sexes **(D)**, females **(E)**, and males **(F)**. Right column **(G–I)**: Frontier analysis of self-harm DALYs rates by sex: black boundary line represents the theoretical efficiency frontier of achievable DALYs rates at each country’s SDI level; points denote observed DALYs rates, with color gradients indicating years from light blue (1990) to dark blue (2021). The 15 countries with the largest efficiency gaps (difference between observed and frontier DALYs rates) are labeled. Panels show results for both sexes **(G)**, females **(H)**, and males **(I)**. SII, slope index of inequality; CI, concentration index; SDI, socio-demographic index; DALYs, disability-adjusted life years.

From 1990 to 2021, the SII for DALYs associated with adolescent self-harm showed a downward trend, decreasing from 108.17 (95% CI: 25.84 to 190.50) in 1990 to −17.74 (95% CI: −82.51 to 47.03) in 2021, reflecting a narrowing of absolute inequality and a shift in burden concentration from higher- to lower-SDI settings (Figure 5A). Sex-stratified analysis revealed that the SII for females declined from 38.60 (95% CI: −16.21 to 93.41) to −51.78 (95% CI: −97.05 to −6.52), indicating reversed inequality and greater burden concentration in lower-SDI settings (Figure 5B). For males, the SII decreased from 158.08 (95% CI: 44.39 to 271.77) to 7.75 (95% CI: −77.14 to 92.64), suggesting that inequality among males has substantially weakened (Figure 5C).

CI results aligned with SII trends, indicating persistent burden concentration in lower-SDI settings. The overall CI increased slightly from −0.21 (95% CI: −0.38 to 0.10) in 1990 to −0.20 (95% CI: −0.35 to 0.06) in 2021, indicating a modest mitigation of inequality (Figure 5D). Among females, the CI rose from −0.30 (95% CI: −0.46 to −0.00) to −0.26 (95% CI: −0.40 to −0.00), suggesting a slight reduction in inequality, whereas among males, the CI decreased from −0.13 (95% CI: −0.31 to 0.16) to −0.15 (95% CI: −0.32 to 0.08), indicating a minor increase in relative inequality (Figures 5E,F).

### Frontier analysis (1990–2021)

From 1990 to 2021, substantial disparities in health efficiency related to the adolescent self-harm burden were observed across countries and territories with varying social development levels. High SDI countries and territories such as Greenland, the United States, Canada, and several Pacific Island nations had DALYs rates markedly exceeding their health frontier values, implying substantial unrealized health gains. In contrast, low SDI countries and territories, including Niger and Somalia, had DALYs rates close to the frontier, suggesting that their burdens were relatively aligned with their development status (Figure 5G; Supplementary Table 4). Among females, high SDI territories such as Palau and Niue had DALYs rates notably exceeding expected levels, whereas low SDI territories like Somalia were near the frontier (Figure 5H; Supplementary Table 4). For males, Greenland showed the greatest efficiency gap, and most low efficiency territories were concentrated in the Pacific Islands and Africa (Figure 5I; Supplementary Table 4). Overall, these deviations suggest that higher socioeconomic development does not ensure greater efficiency in reducing adolescent self-harm.

### Attributable risk factors for adolescent self-harm (1990–2021)

From 1990 to 2021, the PAFs of adolescent self-harm related DALYs due to alcohol use, drug use, and temperature extremes increased globally (Figure 2D; Supplementary Table 5). Alcohol use (PAF = 2.82%) and high temperature exposure (PAF = 2.80%) showed the largest rises, underscoring the growing impact of behavioral and environmental risks. By sex, high temperature was the leading risk for females, while high alcohol use ranked first for males (Figures 2E,F; Supplementary Table 5).

### Predictions of self-harm incidence, YLDs, and DALYs in 2035

**Figure 6:**
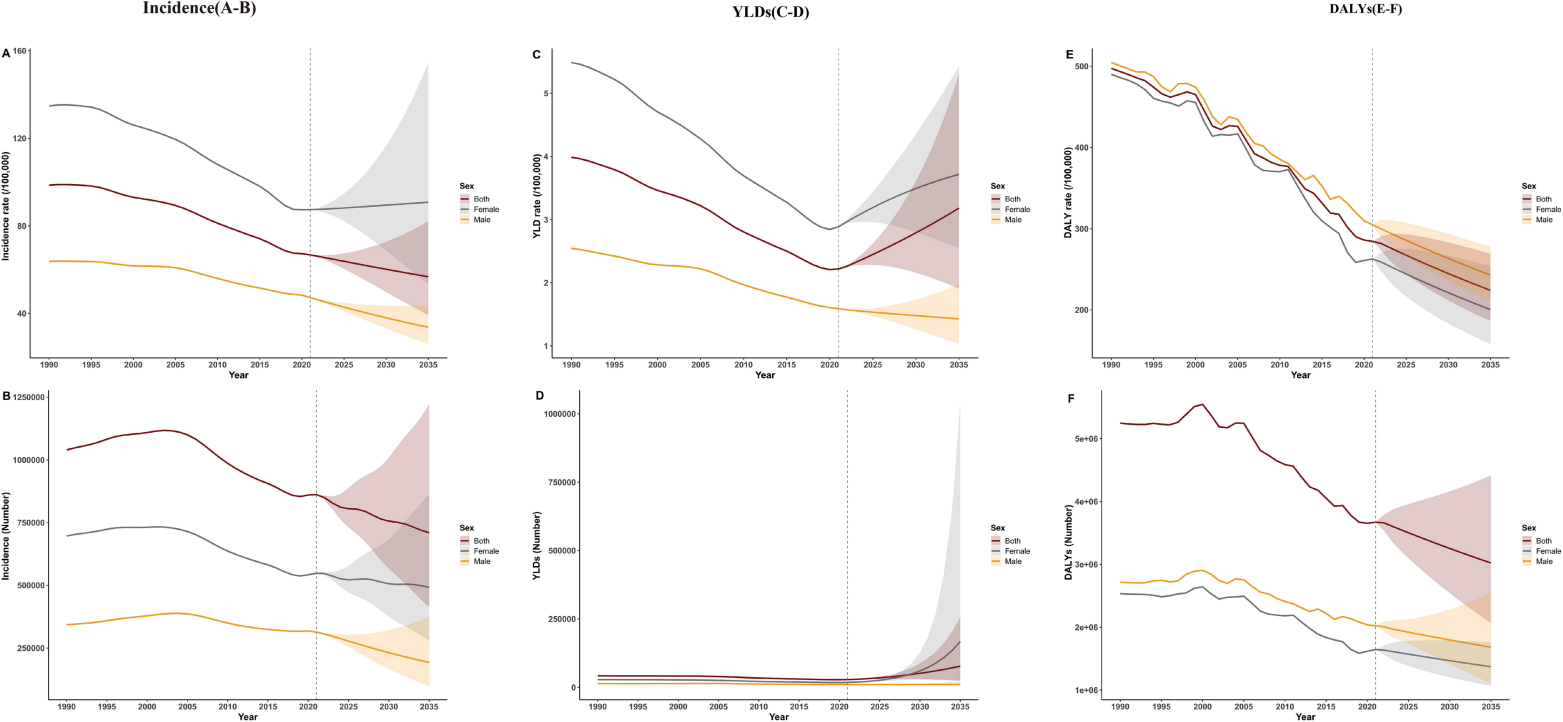
Global projections of adolescent self-harm burden (aged 10–19 years) to 2035. Left column (A, B): Predicted incidence rates **(A)** and numbers of incident cases **(B)**. Middle column (C, D): Predicted YLDs rates **(C)** and numbers of YLDs **(D)**. Right column (E, F): Predicted DALYs rates **(E)** and numbers of DALYs **(F)**. YLDs, years lived with disability; DALYs, disability-adjusted life years.

Based on ARIMA projections, by 2035, global adolescent self-harm incidence is expected to decline overall and among males but rise among females, suggesting widening gender disparities (Figures 6A,B; Supplementary Table 6). YLDs rates are projected to increase, particularly among females (Figures 6C,D; Supplementary Table 6), while DALYs rates are expected to decline across all groups, reflecting reduced fatal burden (Figures 6E,F; Supplementary Table 6).

## Discussion

Our findings revealed worrying heterogeneity beneath the apparent global improvements. Although incidence, YLDs, and DALYs for adolescent self-harm have declined overall, the AAPC in incidence is increasing in nearly 45% of countries and territories. Uzbekistan showed the fastest growth across multiple groups, a pattern consistent with a recent study that noted the Central Asia has one of the highest rates of adolescent self-harm (40). This contrast indicates that global gains in adolescent self-harm prevention are unevenly distributed. The severity of self-harm and its treatment outcomes directly affect mortality and disability burdens (41). While globalization has driven economic and medical progress, it may also bring new risks to adolescents, such as exacerbating social inequality and triggering an identity crisis among them (42, 43). Thus, the benefits of globalization have not translated uniformly into improved psychological resilience, and some populations may disproportionately experience adverse consequences.

We found spatial clustering of adolescent self-harm burden. Greenland, Palau, Kiribati, and other Pacific island nations, as well as areas with extreme environments, constitute the world’s most severe hotspots. While the formation of geographic hotspots may involve a variety of factors, such as environment, culture, historical trauma, and reporting disparities, our findings suggest that environmental and climate vulnerability may play a role independent of socioeconomic status. For example, Greenland’s high rates of self-harm have been documented previously (44) and may relate to extreme cold and polar night contributing to seasonal affective disorder (45). Pacific island nations confront existential threats such as sea-level rise; the resulting collective trauma, displacement pressures, and cultural disruption may together shape a distinct environmental context for adolescent self-harm (46, 47). Therefore, it is recommended that future interventions incorporate geographical and environmental vulnerability factors and integrate climate adaptation into mental health support systems.

From 1990 to 2021, adolescent self-harm burden showed clear sex differences across incidence, health loss, inequality patterns, and attributable risks. Overall, females had higher incidence and YLDs, whereas males had higher DALYs, consistent with GBD evidence and prior studies (13, 16, 48, 49). Our projections suggest female incidence and YLDs will continue to rise, potentially reflecting greater use of self-harm as an emotion-regulation strategy amid affective distress, interpersonal conflict, and violence exposure (50, 51). By contrast, masculinity norms that discourage emotional disclosure may be linked to more lethal methods and a higher DALY burden among males (52–54).

Sex differences also emerged in the relationships between development, inequality, and burden. Female DALY rates were negatively associated with SDI, suggesting socioeconomic development may reduce female burden via improved access to care and stronger social support (55, 56). The reversal in the female SII slope further indicates a potential shift in the burden’s center from higher-SDI toward lower-SDI settings, where economic strain and gendered caregiving responsibilities may heighten psychological stress and barriers to treatment (57, 58). Menstrual stigma, early marriage, and gender-based violence may add risk for females (59, 60). In contrast, male burden showed no significant association with SDI, possibly because service underuse, peer violence, and emotion-regulation difficulties may attenuate any protective effect of development (52, 61–63).

Attributable risks were likewise sex-specific: high-temperature exposure contributed more to the burden among females, whereas alcohol played a larger role among males (64, 65). Heat may increase risk through neuroendocrine dysregulation, impulsivity, and reduced emotion regulation (66, 67), while alcohol-related burden likely reflects higher exposure shaped by cultural norms and behavioral patterns (65). Together, these findings support gender-sensitive prevention that addresses upstream socioeconomic and environmental stressors while tailoring interventions, strengthening emotional support and trauma-informed care for females, and reducing stigma, restricting access to lethal means, and addressing substance misuse among males (68, 69).

Our SDI findings reflect associations rather than causality. The higher incidence in high-SDI settings likely stems from improved surveillance, underlying economic stress, higher mental-disorder prevalence, and adolescent vulnerability, even as stronger health systems may mitigate non-fatal outcomes, explaining why incidence rises with SDI while YLDs decline (54, 55, 58, 70–72). This aligns with the “vulnerability paradox,” where socioeconomic progress does not uniformly improve mental health (73). Conversely, low-SDI settings may retain protective community and family support that buffer risk despite limited resources (74, 75). Although declining SII and CI for DALYs from 1990 to 2021 suggest reduced overall inequality, the burden remains concentrated in lower socioeconomic groups (76). Countries in economic transition often face lagging investments in education and health, weak social protection, and limited mental health coverage, heightening adolescent vulnerability (77–79) while household economic strain, parental mental health issues, and perceived social inequity further elevate risk (80). Thus, prevention must extend beyond economic growth to prioritize equitable mental health access and social protection, especially in resource-constrained settings.

Caution is warranted in interpreting the observed associations between alcohol use, drug use, extreme temperature exposure, and rising self-harm burden, as these are based primarily on observational data. GBD-derived attributable fractions are estimated from observational literature, and complex bidirectional relationships between these risk factors and self-harm behaviors complicate causal attribution. Nevertheless, between 1990 and 2021, attributable burden from alcohol use, drug use, and extreme temperature exposure increased, reflecting a compound behavioral and environmental challenge to adolescent mental health. Despite the implementation of drug abuse control policies in many countries, the resulting burden has not decreased significantly (81, 82), suggesting that individual-level risk management alone is insufficient. Moreover, evidence indicates that relative risk for self-harm and aggressive behaviors increases nearly linearly with temperature (83). Schools should strengthen education on drug abuse prevention and climate adaptation, while communities should establish early warning systems and policies to restrict adolescents’ access to alcohol and drugs.

Global projections indicate an increase in YLDs due to adolescent self-harm by 2035, suggesting that the burden may further shift from mortality toward long-term disability. This trend reflects that more adolescents may experience lasting functional impairment after self-harm (84). It may because a growing number of young people will face long-term psychological, social, and functional challenges in learning, social interaction, and daily life. Even if advances in acute care may reduce fatalities or improve survival, underlying drivers such as emotional distress, trauma, and unmet psychological needs may persist or even intensify (85, 86). These projections underscore the need for health systems to shift from a “crisis-response” model toward one that emphasizes “early prevention and continuous care,” for example, by strengthening early identification, standardized referral, and crisis support in schools and communities.

Although several GBD-based studies report declining self-harm burden in the broader 10–24 age group (2, 16, 87) our focus on 10–19 years reveals more granular epidemiological patterns. First, the finding that 44.61% of countries and territories have rising incidence AAPC highlights concealed heterogeneity masked by global averages, complementing broader-age analyses (16). Second, our projections of rising YLDs and female incidence align with some recent forecasts (13), but diverge from others that predict declines for the wider 10–24 cohort (16), underscoring a potential intensification of nonfatal burden and widening sex differences among adolescents. Third, although some studies suggest protective effects of low temperature (9), our observation of high burden in extreme-environment settings such as Greenland suggests that low temperature may operate as a risk factor under particular conditions. Finally, the “health-efficiency gaps” we identified in high-SDI countries and territories corroborate recent findings on unrealized potential in alcohol-related self-harm control in developed settings (88).

This study has several limitations. First, there are discrepancies in GBD data reporting. Monitoring systems in some low- and middle-income countries are inadequate, self-harm incidents are not included in statutory reporting, or there is insufficient identification at the grassroots level, which may lead to systematic underreporting and thus an underestimation of the actual burden. Second, cultural and social stigma influences the concealment of self-harm. In some societies, self-harm is still considered a moral taboo (89), and this “silent stigma” may cause victims and their families to conceal their actions (90), affecting data accuracy and contributing to apparent regional differences. Third, although the GBD model alleviates the data sparsity problem to some extent through stratified modeling, model smoothing may underestimate the risk level of localized high-incidence areas or short-term fluctuations. National-level aggregated data may mask inequalities within subgroups, such as urban–rural, ethnic, or conflict-affected areas. Therefore, the results are more applicable to macro-trend analysis than absolute comparisons of burdens between countries. Fourth, regarding prediction, although the ARIMA model fits well in the AIC/BIC assessment and the residuals pass the Ljung-Box test to support model robustness, the KPSS test indicates a slight non-stationarity in the series. This characteristic is common in long-term health trend analysis and may reflect the structural impact of socioeconomic fluctuations. The results should be understood as scenario-based predictions under existing trends, rather than definitive conclusions. Fifth, the ARIMA model struggles to capture the “breakpoint effect” of sudden public health events or major policy interventions, limiting the accuracy of long-term extrapolation. Sixth, while further segmentation of adolescents into age groups is crucial for a deeper understanding of disease patterns, this may not be feasible in this study. Because the available age grouping data from 2021 is no longer fully accessible, we were unable to obtain age grouping data suitable for reanalysis. Therefore, this study used the overall 10–19 year age group. Future access to more detailed age grouping data may allow for further exploration of finer age-band differences within adolescents. Finally, this study is based on national data and cannot assess individual-level mechanisms or subgroup heterogeneity. Future research could leverage multinational, individual-level adolescent survey datasets, such as the Health Behavior in School-aged Children (HBSC) study, to examine individual-level determinants and within-country subgroup disparities (91).

To address these challenges, future work should (1) strengthen surveillance capacity in low- and middle-income countries and island territories to improve data representativeness; (2) adopt mixed-methods approaches and bias-correction techniques to adjust for reporting bias; (3) develop multi-source integrated models to enhance predictive accuracy.

## Conclusion

Based on data from the GBD 2021, this study comprehensively analyzed the spatiotemporal trends of adolescent self-harm from 1990 to 2021 and projected future patterns to 2035. The results revealed that although the global overall burden declined, 44.61% of countries and territories still showed increasing annual incidence rates, indicating uneven progress worldwide. Females bore a higher non-fatal burden (YLDs), whereas males experienced a greater fatal burden (DALYs). Some high-SDI countries and territories exhibited a health efficiency gap, while low-SDI countries and territories continued to face high risks. Attributable risks from alcohol use, drug use, and extreme temperature exposure have continued to rise. Forecasts suggest a shift of the self-harm burden toward disability rather than mortality. Policymakers should integrate adolescent mental health screening into school and public health systems and enhance psychological support in climate-vulnerable areas. Clinical and public health efforts should prioritize gender-sensitive care, early detection, and substance-use prevention to reduce self-harm risk and promote mental health equity.

## Data Availability

The original contributions presented in the study are included in the article/Supplementary material, further inquiries can be directed to the corresponding author.

## Footnotes

1
https://vizhub.healthdata.org/gbd-results/
