# Supplementary material for: Global adolescent self-harm (10–19 years): 1990–2021 trends, health inequalities, frontier analysis, and 2035 projections using global burden of disease data
Source: Front Public Health. 2026 Feb 3;14:1689706. doi: 10.3389/fpubh.2026.1689706 (PMC12909592; doi:10.3389/fpubh.2026.1689706)
Supplement: Supplementary file 1 [file Data_Sheet_1.DOCX]

**Appendix: Software and Parameter Settings**

All analyses were conducted in R software (version 4.4.2, R Foundation for Statistical Computing, Vienna, Austria). Detailed software packages and parameter settings used in the analyses are described below.

**1. Time Trend Analysis (AAPC Estimation)**

Package: segmented (v1.6-4)

Model: Generalized linear model with the natural logarithm of age-standardized rate as the dependent variable and year as the independent variable.

Segmentation: Up to 4 breakpoints were allowed; model selection was based on the Bayesian Information Criterion (BIC).

Computation: AAPC was calculated as the weighted average of the APCs across all segments, weighted by segment length.

Confidence Intervals: 95% CIs were estimated using the Delta method to account for sampling and model uncertainty.

Significance Criterion: A trend was considered statistically significant when the 95% CI did not include zero (P < 0.05).

**2. Time Series Forecasting (ARIMA)**

Package: forecast (v8.21)

Model Selection: Parameters (p, d, q) were optimized automatically using the auto.arima() function based on AIC/BIC minimization.

Forecast Horizon: 2022–2035.

Confidence Level: 95%.

Diagnostics: Residuals were tested with the Ljung–Box test (P > 0.05) for independence and the KPSS test (P ≈ 0.01) for stationarity evaluation.

**3. Frontier Efficiency Analysis**

Package: frontier (v1.1-2)

Model Type: Stochastic frontier analysis (SFA).

Distributional Assumption: Truncated-normal error term.

Efficiency Definition: The ratio of observed DALY rate deviation relative to the estimated efficiency frontier.

**4. Health Inequality Analysis**

Packages: ineq (v0.2-13), reldist (v1.7-2)

Indices: Slope Index of Inequality (SII) and Concentration Index (CII).

Uncertainty Estimation: Bootstrap resampling (1,000 iterations).

Interpretation: Results with 95% CI crossing zero were treated as statistically uncertain.

**5. Visualization and Mapping**

Packages: ggplot2 (v3.5.1), sf (v1.0-14)

Purpose: Data visualization and geographical mapping of rates and trends.
